# Post-COVID-19 complications in home and hospital-based care: A study from Dhaka city, Bangladesh

**DOI:** 10.3389/fresc.2022.1037649

**Published:** 2022-11-24

**Authors:** Salamat Khandker, Aivee Akther, Billal H. Syed, Rezoun Shafiullah, Kawsar Ahmed, Alauddin A. Chowdhury, Salim Khan

**Affiliations:** ^1^ Department of Public Health, Daffodil International University, Dhaka, Bangladesh; ^2^ School of Mathematics & Statistics, Central South University, Changsha, China; ^3^ Head, Department of Public Health, Birmingham City University, Birmingham, United Kingdom

**Keywords:** COVID-19 complications, Bangladesh, hospital, home treatment

## Abstract

A cross-sectional survey was undertaken to understand the management patterns and post-COVID-19 complications among hospital and home-treated participants. Retrospective information was collected from four COVID-19 dedicated hospitals and four selected community settings. Using probability proportional sampling, 925 participants were selected. Data were collected using a semi-structured questionnaire. Bivariate and multivariate logistic regression analysis and the exact chi-square tests were utilized to analyze the association between the studied variables. A total of 659 participants responded (response rate 70.93%); 375 from hospitals and 284 from communities. About 80% of participants were mild cases, 75% were treated at home, and 65% of hospital-treated participants were referred after home treatment. Participants treated at home-to hospital and directly in the hospital had 1.64 and 3.38 times longer recovery time respectively than what home-based participants had. A significant increasing trend (*p* < 0.001) of co-morbidities was found among referred and hospital treated participants. Age, level of education, physical exercise, practicing preventive measures, exposure to sunlight, and intake of carbohydrate, additional liquid, food supplements, and avoidance of junk foods were significantly associated with place of treatment. Post-COVID-19 difficulties of all factors were statistically significant for home treatment participants, whilst only depression (*p* = 0.026), chest pain (*p* = 0.017), and digestive disorders (*p* = 0.047) were significant (*p* < 0.05) for hospital treated participants. The outcomes from this study provide insight into a range of post-COVID-19 difficulties relating to at home and in hospital treatment participants. There are clear differences in the complications experienced, many of which are statistically significant. The health care professionals, the community people and COVID-19 survivors will be benefitted from the study findings, and the policy level people may use the information for designing health education program on post COVID-19 complications.

## Introduction

Bangladesh is one of the most densely populated countries in the world with a population density of 1,126 per km^2^ (1). In 2020 per capita, Gross Domestic Product (GDP) was 1,968.79 US dollars, and 20.5% of the population lived below the national poverty level in 2019 (2). The government health expenditure is about 34% of the total health cost and the remaining 66% being out of pocket expenses. Bangladesh does not have a comprehensive health policy to strengthen the entire health system. The country has a growing private sector health care services mainly providing tertiary level care (3). In 2016, the number of hospital beds per 1,000 people was 0.8 (4). Only about 3% of the GDP is spent on health services (5). This study was carried out in the capital city Dhaka which has a population of 8,906,000 (1).

The Ministry of Health and Family Welfare (MOH&FW) is the Government authority charged with policy, planning, regulatory and decision-making in the health service sector. There are four Directorates work under MOH&FW, namely, the Directorate General of Health Services, (DGHS), Directorate General of Family Planning (DGFP), Directorate General of Nursing and Midwifery (DGNM), and Directorate General of Drug Administration (DGDA) (6). The Director General of Health Services is the watchdog for health care delivery services throughout the country. Bangladesh is undergoing a health transition and manifesting a double burden of disease attributable to non-communicable diseases with existing communicable diseases. The government, private sector, non-government organizations (NGOs), and, donor agencies are playing the main role in the health service system of Bangladesh. There are 12,727 community clinics that facilitate bringing health services to the doorsteps of the people. The private sector consists of the formal and informal sectors. The formal sector consists of western and traditional services with trained service providers and informal sectors with untrained service providers. The private services are poorly regulated (7). The healthcare sector of the country reached USD 6.76 billion in 2018 and is dominated by the private sector with high growth in tertiary level care. There are 255 public hospitals, 5,054 private hospitals and clinics, and 9,529 diagnostic centers under the registration of the DGHS. The total number of hospital beds in 2019 was 143,394 among these 45,660 were in the public sector and 91,537 were in the private sector (8). In April 2020 Bangladesh government declared the names of several private and public hospitals as COVID-19 dedicated hospitals in the country. Later the services for COVID-19 patients were extended to all tertiary-level health care facilities, district hospitals (secondary-level care), and some Upazila Health Complexes (Primary-level care) (9).

The World Health Organization (WHO) declared COVID-19 a pandemic on March 22, 2020 (10) From 3 January 2020 to 14 October 2022, there were 2,031,797 confirmed cases of COVID-19 in Bangladesh, with 29,393 deaths reported to WHO (11) Numerous clinical studies reveal that high proportions of hospitalized COVID-19 patients continue to suffer from one or more health complaints for months after discharge (12, 13). A working group under WHO has recently proposed a preliminary clinical case definition using the term “Post COVID-19 condition”. This definition is based on a structured consensus process that is present three months after the onset of symptoms or date of SARSCoV2 infection which lasts at least two months (14–17).

Multiple research have demonstrated that 50 to 87% of hospitalized patients globally experienced at least one post-COVID-19 symptom for several weeks following convalescence or hospital discharge, while 20% of COVID-19 patients experienced persisting symptoms for more than three months although the severity was diminished (18–20). Such persistent and debilitating symptoms following COVID-19 indicate that the negative consequences of the outbreak did not end with recovery from the acute phase of the disease and that the pandemic is likely to have an ongoing impact on individuals, families, and social groups (21). More than 80% of COVID-19 patients need home-based care, while less than 20% need hospital admission and receive therapies ranging from oxygen to ventilator support (22, 23). In Bangladesh, the patients admitted to hospitals were administered analgesics to alleviate pain, drugs to control fever, oxygen for managing respiratory distress, saline for maintaining proper hydration, and additional antibiotics, antiviral drugs, and plasma therapy were concurrently given where deemed (23). A tendency of eating garlic, lime and lemons with food, taking a hot gurgle, hot water bath and sunbathing were performed by the individual to get rid of pandemic. However, maintaining social distance, wearing musk, keeping etiquette during sneezing, and self-quarantine at home or institutional isolation are the recommended ways to manage mild or moderate cases. This study was aimed to evaluate preventive measures taken by the respondents, ascertain treatment and recovery procedures, and identify patterns of food intake during the process of recovery in patients who have received home and hospital-based care.

## Materials and methods

A cross-sectional survey was undertaken to understand the pattern of COVID-19 management and prevention in urban Bangladesh. The study population included COVID-19 positive patients treated at home or in a hospital who were 18 years or above regardless of gender, religion, ethnicity, level of education, or occupation. Patients attending the selected hospitals from other cities were not included in the study. Four hospitals (Dhaka Medical College Hospital, Magda Medical College Hospital, Shaheed Suhrawardy Medical College Hospital, and Kurmitola General Hospital) were randomly selected from the list of COVID-19 dedicated hospitals in Dhaka city. In addition, four wards from each North and South City corporation of Dhaka were randomly selected for community level data collection.

A total of 925 COVID-19 positive cases using the probability proportional sampling procedure were selected; starting from the recent discharge date and proceeding backwards until the required number was attained. Contact was made *via* the phone with individual patients where survey objectives were clearly explained. Upon receiving verbal consent, the individuals were invited to participate in the survey. A total of 659 patients completed the survey, 375 from hospitals and 284 from community settings. Data were collected by both Electronic Survey Form and face-to-face interview using a semi-structured questionnaire. A multidisciplinary team including social scientists, epidemiologists, physicians, and statisticians were involved in developing the questionnaire. The questionnaire was pre-tested in non-sampled participants in the hospitals and community wards. Field staff were intensively trained on the background of the study, objectives, methodology, and interviewing techniques. Before the interview, an informed written/verbal consent was taken once more from the participants. Data were re-checked, coded, and entered into a database using SPSS software. Incorrect/incomplete data were rejected. Key findings were articulated with descriptive statistics. Bivariate and multivariate logistic regression analysis was used to determine association between the level of treatment, management, and preventions with participant's demographic characteristics and other factors. Participant's pattern of COVID-19 treatment was considered a dependent variable and used as a reference category. Covariates considered in the modeling included age, sex, socio- demographic status, use of protective measures, place of management, care and other factors. To investigate the relationship between treatment location and the degree of post-COVID-19 complications, adjusted bivariate logistic models were run for the two-response variable such as hospital-based treatment and home-based treatment using Statistical Package for Social Sciences (SPSS version 26). The current study used models with binary outcomes, a variety of post-COVID-19 symptoms. Covariates were coded as no and yes, and adjusted variables were gender, marital status, and comorbidities. To analyze the associations between the studied variables, we performed *χ*^2^ test, but in some cases where the number of option was less than 5; Fisher's exact test was performed. The odds ratio was estimated with a 95% confidence level for the coefficient impact at 5% level of significance.

In this study participants having an academic qualification above fourteen years were considered as bachelor. Body Mass Index (BMI) between 18.5 to less than 25 was considered as healthy weight. While BMI less than 18.5, between 25 and 30, and above 30 were considered as underweight, overweight and obese respectively.

## Results

Socioeconomic and demographic characteristics of respondents are presented (Table 1) to facilitate in comparing findings with similar characteristics in other independent variables. The data shows that of the study participants, 40% were male and 60% were female. The mean age of participants was 38.43 ± 13.90, and 46% had a bachelor's degree or higher qualification. Around 42% were service holders and the median monthly family income was Bangladeshi Taka (BDT) 50,000.00. The mean (±SD) family size was 4.42 (±1.39). Some 10% of participants were obese, whilst 6% were found to be underweight. Of the study participants, 62% did not suffer any comorbidity. Diabetes (23%), cardiovascular disease (20%), asthma/Chronic Obstructive Pulmonary Disease (COPD) (8%), rheumatoid arthritis (4%) and Chronic Kidney Disease (CKD) (2%) were prevalent in those with comorbidities.

**Table 1:** Distribution of participants according to socio-demographic characteristics, body mass index, comorbidities and severity of disease.

Background characteristics	Number	Percent
Gender		
Male	262	39.8
Female	397	60.2
Age (in years)		
Up to 29	195	29.6
30–49	312	47.3
50 and above	152	23.1
Mean ± SD	38.43 ± 13.90	
Level of education		
Up to Secondary	168	25.5
Higher Secondary	188	28.5
Bachelor & above	303	46.0
Marital status		
Single	136	20.6
Married	523	79.4
Occupation		
Health service provider	154	23.4
Service	278	42.2
Business	79	12.0
Housewife	124	18.8
Student	24	3.6
Monthly family income (Bangladeshi Taka)		
Up to 50,000	379	57.5
>50,000	280	42.5
Mean, Median	54,633, 50,000	
Family size Mean ± SD	4.42 ± 1.39	
Body Mass Index category		
Underweight	37	5.6
Normal weight	423	64.2
Overweight	131	19.9
Obese	68	10.3
Comorbid conditions		
None	407	61.8
Diabetes	151	22.9
Cardiovascular disease	130	19.7
Asthma/COPD	52	7.9
Rheumatoid Arthritis	25	3.8
Chronic Kidney Disease (CKD)	11	1.7
Others	19	2.9
Severity of cases		
Mild case	524	79.5
Severe case	135	20.48

**Figure 1:**
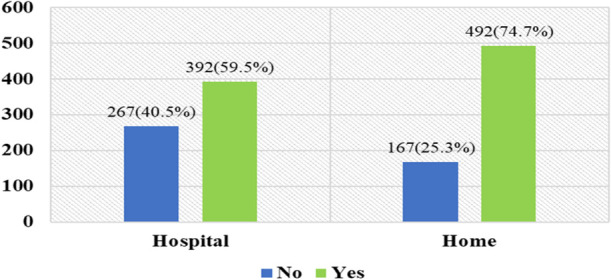
Home and hospital-based treatment.

After testing positive for COVID-19, participants were either treated at home (75%) or in hospitals (60%) (Figure 1). Of the 392-hospital treated COVID-19 participants, 139 (36%) went to hospital directly whilst 253 (65%) were referred after home treatment by a qualified physician at the initial stage of infection.

Mild COVID -19 infection was present in 80% of participants; among which 56% were treated at hospital and 44% at home. For those with severe infection, 73% were treated in hospital and 27% referred to hospital after primary treatment at home. Hence 59% of participants required hospital treatment at some stage (Figure 1). Interestingly, the data also revealed that 69% of home-based treated patients recovered within 14 days, and of those who were in hospital, 54% of referred cases and 39% of directly admitted cases recovered within 14 days (Table 2). A significant finding was that around 61% of the directly admitted participants required more than 14 days to recover whilst only 46% referred cases and 31% of home-treated patients required more than 14 days recovery time. Self-hospitalized patients required more time to recover compared to home based treated and referred cases (*p* = 0.000).

**Table 2:** Distribution of participants by place of treatment and association with characteristics of interest.

Characteristics of interests	Place of treatment			Total (*N* = 659)	Chi-square Test	
	Home (*n* = 267)	Home to Hospital (*n* = 253)	Hospital (*n* = 139)			
	Number (%)	Number (%)	Number (%)	Number (%)	Value	*p*-value
Age						
Up to 29	101 (51.8)	63 (32.3)	31 (15.9)	195 (29.59)	31.956	<0.001
30–49	132 (42.3)	113 (36.2)	67 (21.5)	312 (47.34)		
50 & above	34 (22.4)	77 (50.7)	41 (27.0)	152 (23.06)		
Level of education						
Up to Secondary	61 (36.3)	80 (47.6)	27 (16.1)	168 (25.49)	29.785	<0.001
Higher Secondary	62 (33.0)	64 (34.0)	62 (33.0)	188 (28.52)		
Bachelor & above	144 (47.5)	109 (36.0)	50 (16.5)	303 (46.73)		
Do any form of physical exercise						
No	114 (34.5)	111 (33.6)	105 (31.8)	330 (50.07)	45.760	<0.001
Yes	153 (46.5)	142 (43.2)	34 (10.3)	329 (49.92)		
Preventive Measures taken						
Never	12 (57.1)	5 (23.8)	4 (19.0)	21 (3.1)	22.442	<0.001
Sometimes	64 (29.1)	93 (42.3)	63 (28.6)	220 (33.38)		
Always	191 (45.7)	155 (37.1)	72 (17.2)	418 (63.42)		
Had 15–20 min of sunlight every day						
No	131 (39.9)	144 (43.9)	53 (16.2)	328 (49.77)	12.752	0.002
Yes	136 (41.1)	109 (32.9)	86 (26.0)	331 (50.22)		
Recovery time and place of treatment						
Up to 14 days	184 (68.91)	136 (53.75)	54 (38.84)	374 (56.75)	35.168	0.000
Above 14 days	83 (31.08)	117 (46.25)	85 (61.49)	285 (43.25)		
Co-morbidities among respondents						
None	197 (73.78)	156 (61.66)	54 (38.84)	407 (61.8)	85.848	<0.001
At least 1	56 (20.97)	58 (22.92)	30 (28.77)	144 (21.85)		
At least 2	10 (3.74)	28 (11.06)	44 (31.69)	82 (12.44)		
Three or more	4 (1.49)	11 (4.34)	11 (7.91)	26 (3.94)		
Number of Post COVID-19 complications						
None	76 (28.46)	41 (16.20)	77 (55.39)	194 (29.43)	71.365	<0.001
At least one	22 (8.23)	25 (9.88)	9 (6.47)	56 (8.49)		
At least 2	24 (8.98)	42 (16.60)	9 (5.77)	75 (11.38)		
Three or more	145 (54.30)	145 (57.31)	44 (31.65)	334 (50.58)		

Participants treated at home-to-hospital and those treated directly at the hospital were 1.64 (CI, 1.10–2.42) and 3.58 (CI, 2.13–6.00) times more likely to have longer recovery time respectively, compared with those treated at home. This finding clearly indicates that patients treated at home in the very early stages of their COVID-19 infection did not require a visit to a hospital, or, if needed, were less likely to stay longer in hospital when compared to patients who visited a hospital without having any treatment at home.

The selected characteristics of respondents were investigated (Table 2) to assess the behavior of study participants by place of treatment whilst infected. Age, level of education, performing physical exercise, practicing preventive measures, and daily exposure to sunlight were significantly associated with place of treatment. Furthermore, among the home-treated patients, 74% did not have any co-morbidity and showed reducing trend towards home treatment with increased number of comorbidities. However, the percentage of referred and hospital treated cases showed significant increasing trends with increased number of comorbidities (*p* < 0.001). Table 2 further shows that post-COVID-19 complications found among the patients based on their place of treatment were very significant. Patients treated at home, home to hospital, and directly in hospital having none of the complications were 28%, 16% and 55% respectively. On the other hand, having three or more complications were 54%, 57% and 32% respectively. In total, 29% of participants did not have any complications and 51% had at least 3 post-COVID-19 complications.

The prescribed medicines used for participants (Table 3) were antipyretics (87%), antihistamine (83%), antiallergics (81%), antibiotics (75%), vitamins (66%) and antivirals (37%) Moreover, 23% hospital treated participants received oxygen. Among severe cases 51%, mild cases 28%and home treatment cases 2% received oxygen therapy. An average of 2% of patients required ventilation and ICU support (severe case 5% and mild cases 3%). Of the mild cases, a comparatively higher percentage of home treated patients were given antipyretics, antiallergic, antihistamine, vitamins, antibiotic, antiviral, and antifungal drugs. Around 3% of participants received plasma therapy and all of these were hospital treated.

**Table 3:** Prescribed medicine used at home and hospital-based management (*n* = 659, multiple response).

Type of treatment	Mild case (*n* = 524)		Severe cases (*n* = 135)	Total (*n* = 659)
	Hospital *n* = 294 Number (%)	Home *n* = 230 Number (%)	Hospital^a^ *n* = 98 + 37 Number (%)	Number (%)
Oxygen (low, medium, high flow)	81 (27.55)	4 (1.73)	69 (51.1)	154 (23.36)
Antipyretic, Paracetamol, ibuprofen	272 (92.20))	215 (93.47)	87 (64.4)	574 (87.10)
Antiallergic, Montelukast, Doxophylline, Theophylline	242 (82.3)	210 (91.3)	85 (62.6)	537 (81.48)
Antihistamine (Fexofenadine, Cetirizine)	245 (83.05)	215 (93.47)	86 (63.7)	546 (82.85)
Antibiotics (Doxycycline, Azithromycin)	219 (74.48)	211 (91.73)	67 (49.62)	497 (75.41)
Antivirals (Remdesivir)	86 (29.25)	118 (51.30)	38 (28.14)	242 (36.72)
Antifungals (Ivermectin)	49 (16.66)	42 (18.26)	21 (15.55)	112 (16.99)
Treatment employed at Hospital—Steroids (Dexamethasone, Methylprednisolone)	68 (23.12)	00.00	21 (15.55)	89 (13.50)
Heparin (LMWH)	53 (18.02)	16 (6.95)	15 (11.11)	84 (12.74)
Immunotherapy (Tocilizumab)	6 (2.04)	00.00	5 (3.70)	11 (1.66)
Vitamins (A, B, C, D, E)	186 (63.26)	193 (83.91)	57 (42.22)	436 (66.16)
Plasma therapy	17 (5.78)	0.00	3 (2.22)	20 (3.03)
Ventilator/ICU Support	8 (2.72)	0.00	7 (5.18)	15 (2.27)
Others	33 (11.22)	32 (13.41)	5 (3.70)	70 (10.62)

^a^ Total 37 severe cases were treated at home and were referred to hospital for further treatment.

Food intake behavior, the intake of carbohydrates, additional liquid intake, food supplements, and avoidance of junk foods were significantly associated with the place of treatment sought and demonstrated less hospitalization due to severity (Table 4).

**Table 4:** Distribution of participants by place of treatment and food intake (*n* = 659).

	Place of treatment			Total	Chi-square Test	
	Home Number (%)	Home to Hospital	Hospital			
		Number (%)	Number (%)	Number (%)	Value	*p*-value
Carbohydrate intake increased						
No	50 (36.5)	38 (27.7)	49 (35.8)	137	23.460	<0.001
Yes	217 (41.6)	215 (41.2)	90 (17.2)	522		
Protein intake increased						
No	2 (28.6)	1 (14.3)	4 (57.1)	7	5.679	0.058
Yes	265 (40.6)	252 (38.7)	135 (20.7)	652		
Vitamins & Minerals intake increased						
No	0 (.0)	1 (100.0)	0 (.0)	1	1.607	0.448
Yes	267 (40.6)	252 (38.3)	139 (21.1)	658		
Drunk 8–10 glasses of liquid (water, soup, herbal tea, fruit juice, etc.) every day						
No	33 (62.3)	16 (30.2)	4 (7.5)	53	12.752	0.002
Yes	234 (38.6)	237 (39.1)	135 (22.3)	606		
Took any food supplement						
Nos	144 (47.2)	123 (40.3)	38 (12.5)	305	26.905	<0.001
Ye	123 (34.7)	130 (36.7)	101 (28.5)	354		
Avoided junk food (Fast food), preserved food						
No	68 (48.9)	64 (46.0)	7 (5.0)	139	27.289	<0.001
Yes	199 (38.3)	189 (36.3)	132 (25.4)	520		

**Figure 2:**
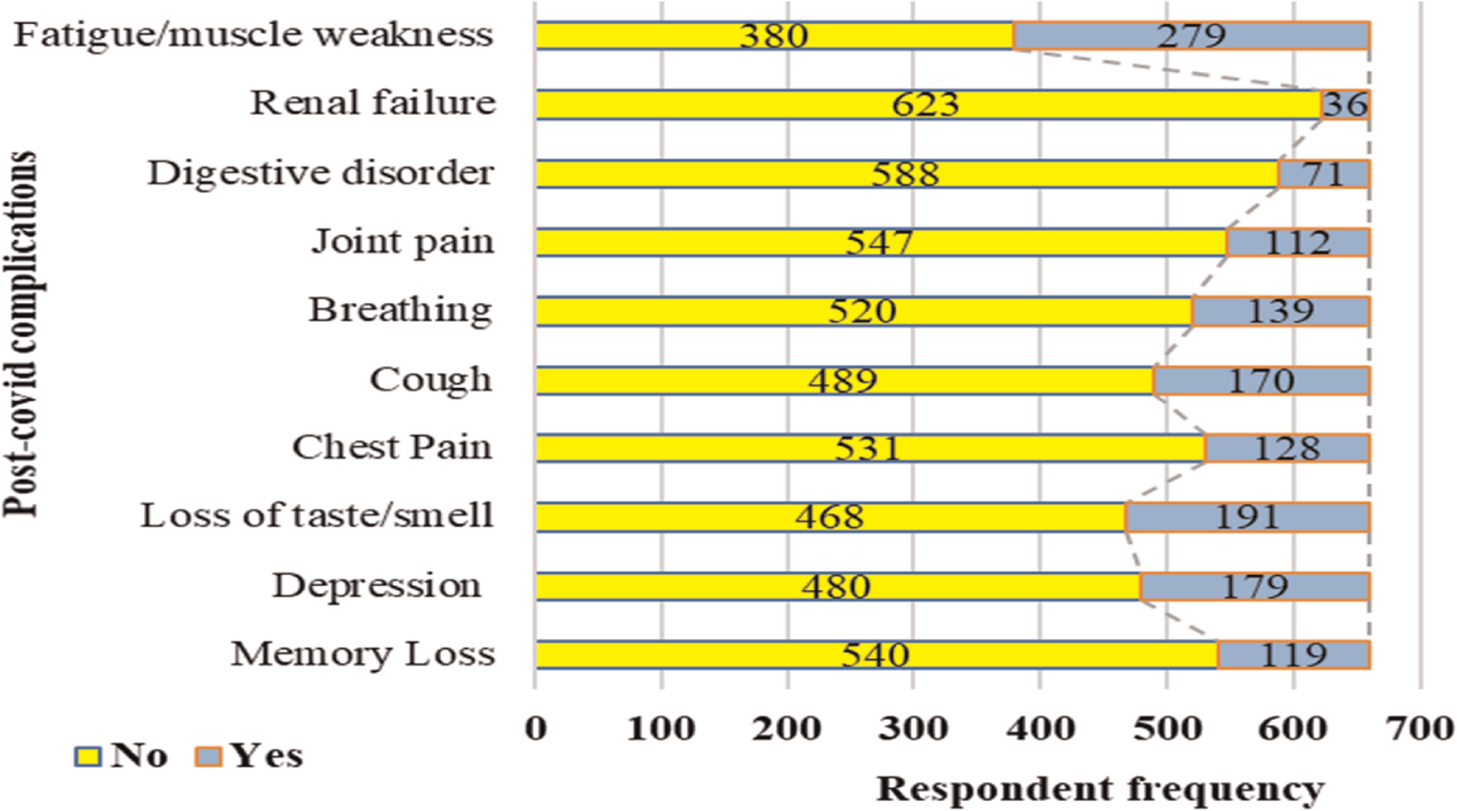
Association between post COVID-19 complications and symptoms.

A range of post-COVID-19 symptoms were identified in participants regardless of treatment at home or in hospital (Figure 2). These include muscle weakness (42%), renal failure (6%), digestive disorders (11%), joint pain (17%), breathing problems (21%), dry cough (26%), chest pain (19%), loss of appetite or smell (29%), depression (27%), and symptoms of memory loss (18%). Other symptoms included headache, blurred vision, palpitation, tremors, and skin infections. The post-COVID-19 difficulties of all factors were statistically significant for those who received home care, while depression (*p* = 0.026), chest pain (*p* = 0.017), and digestive disorder (*p* = 0.047) were only associated significantly at the 5% level (*p* < 0.05) for those who received hospital treatment.

Chi-square test (Table 5) reveals a significant association between post-COVID-19 complications like depression, chest pain, digestive disorder with hospital-based treatment. For those treated at home, memory loss, loss of taste/smell, chest pain, cough, breathing difficulty, joint pain/arthralgia, digestive disorder, fatigue/muscle weakness were significantly associated.

**Table 5:** Post-COVID-19 complications association with home and hospital-based care.

Post-COVID Complications	Hospital-based treatment			Home-base treatment		
	No *n* (%)	Yes *n* (%)	(*χ*^2^) *p*-value	No *n* (%)	Yes *n* (%)	(χ^2^) *p*-value
Memory Loss						
No	212 (32.2)	328 (49.8)	(1.96) 0.16	149 (22.6)	391 (59.3)	(8.01) 0.005
Yes	55 (8.3)	64 (9.7)		18 (2.7)	101 (15.3)	
Depression						
No	182 (27.6)	298 (45.2)	(4.95) 0.026	132 (20)	348 (52.8)	(4.35) 0.036
Yes	85 (12.9)	94 (14.3)		35 (5.3)	144 (21.9)	
Loss of taste/smell						
No	185 (28.1)	283 (42.9)	(0.651) 0.42	159 (24.1)	309 (46.9)	(63.6) 0.000
Yes	82 (12.4)	109 (16.5)		8 (1.2)	183 (27.8)	
Chest pain						
No	227 (34.4)	304 (46.1)	(5.65) 0.017	147 (22.4)	384 (58.3)	(7.92) 0.005
Yes	40 (6.1)	88 (13.4)		20 (3.1)	108 (16.4)	
Cough						
No	205 (31.1)	284 (43.1)	(1.56) 0.212	140 (21.2)	349 (53)	(10.83) 0.001
Yes	62 (9.4)	108 (16.4)		27 (4.1)	143 (21.7)	
Breathing difficulty						
No	217 (32.9)	303 (46)	(1.51) 0.219	156 (23.7)	364 (55.2)	(28.79) 0.000
Yes	50 (7.6)	89 (13.5)		11 (1.7)	128 (19.4)	
Joint pain/arthralgia						
No	215 (32.6)	332 (50.4)	(1.95) 0.162	160 (24.3)	387 (58.7)	(25.99) 0.000
Yes	52 (7.9)	60 (9.1)		7 (1.1)	105 (15.9)	
Digestive disorder						
No	246 (37.3)	342 (51.9)	(3.95) 0.047	162 (24.6)	426 (64.6)	(14.08) 0.000
Yes	21 (3.2)	50 (7.6)		5 (0.8)	66 (10)	
Renal failure						
No	254 (38.5)	369 (56)	(0.307) 0.58	163 (24.7)	460 (69.8)	(4.06) 0.044
Yes	13 (2)	23 (3.5)		4 (0.6)	32 (4.9)	
Fatigue/muscle weakness						
No	161 (24.4)	219 (33.2)	(1.27) 0.258	143 (21.7)	237 (36)	(71.75) 0.000
Yes	106 (16.1)	173 (26.3)		24 (3.6)	255 (38.7)	

Fisher's exact test when more than 20% of cells have expected frequencies less than five. *P*-value (≤0.049).

The outcome of the adjusted odds ratio logistic model for the response of hospital and home-based medical therapy for the variables of post-COVID-19 complications and symptoms assessment were also determined (Table 6). Depression, coughing, and joint pain or arthralgia were statistically significant after therapy in responders who received hospital-based care. Furthermore, memory loss, loss of taste or smell, chest discomfort, and breathing issues were statistically significant in participants who received treatment at home but not in those who received care in hospital. Nevertheless, digestive disorders were significant in participants who received hospital care [OR = 2.04, 95%C.I. = (1.07–3.91), *p* < 0.05] and home treatment [OR = 3.90, 95%C.I. = (1.20–12.63), *p* < 0.05]. Similarly, post complications of fatigue or muscle weakness were significantly allied to hospital treatment [OR = 1.58, 95%C.I. = (1.08–2.31), *p* < 0.05], and home-based care [OR = 5.23, 95%C.I. = (2.99–9.12), *p* < 0.05], respectively.

**Table 6:** Post-COVID-19 complications with hospital and home-based treatment.

Study Post-COVID Characteristics	Model (AOR)							
	Hospital-based treatment				Home-base treatment			
	B	S.E.	*p*-value	95% C.I. OR [Upper-Lower]	B	S.E.	*p*-value	95% C.I. OR [Upper-Lower]
Memory Loss (No vs. Yes)	−0.27	0.24	0.27	0.76[0.46–1.23]	0.04	0.35	0.01	1.05[0.51–2.12]
Depression (No vs. Yes)	−0.63	0.21	0.03	0.53[0.34–0.81]	–0.34	0.29	0.24	0.70[0.399–1.26]
Loss taste/smell (No vs. Yes)	−0.38	0.22	0.08	0.68[0.44–1.04]	2.20	0.42	0.00	9.08[3.91–21.08]
Chest pain (No vs. Yes)	0.45	0.26	0.08	1.5[0.93–2.63]	–0.80	0.38	0.03	0.44[0.209–0.94]
Cough (No vs. Yes)	0.52	0.24	0.03	1.68[1.04–2.72]	–0.44	0.33	0.19	0.64[0.333–1.24]
Breathing difficulty (No vs. Yes)	0.14	0.24	0.57	1.15[0.70–1.87]	1.24	0.41	0.00	3.45[1.54–7.72]
Joint pain/arthralgia (No vs. Yes)	−0.73	0.26	0.00	0.47[0.28–0.80]	0.63	0.46	0.17	1.89[0.75–4.70]
Digestive disorder (No vs. Yes)	0.71	0.33	0.03	2.04[1.07–3.91]	1.36	0.59	0.02	3.90[1.20–12.63]
Renal failure (No vs. Yes)	−0.15	0.43	0.723	0.85[0.371.99]	–0.03	0.72	0.96	0.97[0.23–4.00]
Fatigue/muscle weakness (No vs. Yes)	0.46	0.19	0.02	1.58[1.08–2.31]	1.65	0.28	0.00	5.23[2.99–9.12]

Accepted *p*-value (≤0.049). 95% Confidence Interval (C.I.), Both Model Adjusted Odds Ratio (AOR), Reference No.

## Discussion

Among 659 participants of our study, 39.8% were male and 60.2% were female, which differed from the findings of most previous studies undertaken both in Bangladesh and abroad (24–26) as well as the data given in a systemic review and meta-analysis of COVID-19 clinical features (27). The mean age of the participants was 38.43 ± 13.90, which was similar to three previous studies in Bangladesh (28, 29). Around 42% of the participants were service holders, median monthly family income was BDT 50,000.00. The average family size was 4.42 (±1.39), which is consistent with previous reports (30). According to research conducted in the United States, socioeconomic status, the poverty rate, unemployment rate, per-ca-pita income, educational attainment, and living in crowded housing, is connected with the risk of COVID-19 occurrence (31). Researchers from Bangladesh also found an association between covid-19 patients' educational level and profession (28, 29). About 10% of the participants were obese, 6% were underweight, and 62% had no comorbidity. Among the comorbidity of this study the most prevalent was was diabetes, cardiovascular disease, asthma/COPD, rheumatoid arthritis, and CKD. Other Bangladeshi studies revealed that hypertension, bronchial asthma, and diabetes, were the most common co-morbid conditions among COVID-19 patients (31). By an Iranian study, diabetes and cardiovascular disease were shown to be the most prevalent comorbidity among COVID-19 patients (32).

This study found that females of working age had an increased COVID-19 infection rate, a finding also observed in a German based study (33). A meta-analysis however (34) reported no difference in the proportion of males and females with confirmed COVID-19 but did show that a majority of those infected were of working age.

An interesting finding was that COVID-19 positive individuals who were treated in hospital had longer recovery times. A previous study reported that the average hospital stay of COVID-19 patients was 14.5 days (35) which relates to observations in the current study. Those treated at home during the early phase of COVID-19 infection did not generally require hospitalization; however if needed, had shorter hospital stays compared to COVID-19 positive individuals who attended a hospital without any previous treatment at home.

The participants in the current study who exercised regularly were less likely to need hospital treatment. The benefits of physical activity/exercise are well documented, particularly in terms of improving health outcomes and increasing immune activity (36). Other studies which also reported that physical activity was instrumental in lowering the probability of hospital admission in COVID-19 patients (37); and athletes having a 33% reduced hospitalization rate than non-athletes (38).

Present findings identified that those individuals who were exposed to sunlight whilst receiving treatment in hospitals or at home were more likely to recover from the infection and that daily exposure was significantly associated with place of treatment; this being supported by other researchers (39). This correlation is consistent with previous evidence that sunlight can improve the health of COVID-19 patients as it boosts the immune system (40, 41). There is much discussion regarding the benefits of Vitamin D3 in contributing to COVID-19 prevention (42, 43). Furthermore, the direct effect of solar UV-A/B or artificial UV-C (100–280 nm) light on SARS-CoV-2 inactivation has been documented (44) and with some studies finding a direct link between Vitamin D3 deficiency and COVID-19 (45).

Whilst a range of clinical symptoms were identified, these were higher amongst those with severe COVID-19 infection. Similar findings were identified in a recent study in China where significant differences were observed between those with mild and severe COVID-19 infection (46). Several investigations have suggested that SARS-CoV-2 affects the neurological system and skeletal muscles (47). Observational research in Bangladesh reported that gastrointestinal problems such as anorexia, diarrhea, and abdominal pain were frequent among COVID-19 patients (48). In the current study, post-COVID consequences of digestive illness, fatigue, or muscle weakness were considerable across both groups of respondents who had hospital care and home treatment.

This study revealed that the place of treatment was significantly associated with intake of carbohydrate, food supplements, and additional liquid, and avoidance of junk foods i.e., people treated in a hospital get better and more balanced food intake. The value of food and natural products to help with possible effective preventative and co-therapeutic methods for viral infections is gaining much traction (49). The Food and Agriculture Organization of the United Nations has emphasized the consumption of a healthy and balanced diet for the prevention of COVID-19 (50). Changes in dietary habits contribute to major changes in immunity in older persons and nutrients like omega-3, polyunsaturated fatty acids, and probiotics have increased resistance to upper respiratory tract infection (51). Eating a variety of fresh and unprocessed foods with a limit on sugar, fat and salt is suggested by the WHO and UNICEF during COVID-19 infection (40, 41). Vitamin C and D, as well as zinc and selenium supplementation may also be beneficial for people who are at risk of respiratory virus infections, according to the Brazilian Association of Clinical Nutrition (52).

In this study use of antibiotics, antifungal, histamine and allergic substances, was higher among home treated respondents. Additionally hospital treated participants received immunotherapy, plasma therapy, ventilator use, and steroids. It has previously been reported that significantly higher doses of corticosteroids, remdesivir, and tocilizumab have been used in patients of COVID-hospitals than non-COVID-hospitals (53). In the current study immunotherapy was only administered to severely infected patients who had received hospital treatment. In the United States, monoclonal antibodies are authorized for patients with symptomatic but mild to moderate infection and high-risk group patients (above 65 years age, comorbidities, pregnant women, immunosuppression, sickle cell disease) (54).

The present study reported the frequency of a range of symptoms such as headaches, joint pains, muscle weakness, renal failure, which are consistent with a WHO report (55). In addition, around 70% of participants reported post-COVID-19 complications with some 50% of respondents suffering from several complications. The post-COVID-19 complications of all factors were significantly increased for those who received home care. Although home-treated participants required significantly less recovery time than the participants those were admitted directly to hospital and referred to hospital for admission. On the other hand, in participants who received hospital-based treatment, depression, coughing, and joint pain or arthralgia were significantly elevated following treatments. Previous study reported that extra-pulmonary multi-organ dysfunction, rates of diabetes, major adverse cardiovascular event (MACE), kidney disease, and liver disease were significantly elevated after discharge with COVID-19 patients and that fatigue, myalgia, arthralgia, and reduced physical activities were the most common physical post-COVID complications (56, 57), which is consistent with the current study findings. A longitudinal study may be designed to understand the reason of higher proportion of post-covid complications among home-treated participants and determine the reasons of less recovery time than hospital treated participants.

This study identified significant issues associated with current evidence of treatment pattern, management and preventions. The findings are expected to distinctively explore to what extent people resorted to hospital service and family care in dealing with COVID-19 positive patients. These findings may also help in developing new Behaviour Change Communication (BCC) materials; help the healthcare monitoring and enforcing authorities to set future strategies to combat possible pandemic; or they can be shared among healthcare experts to initiate new policies to improve the health and well-being of COVID-19 survivors.

## Conclusions

This study provides an interesting insight into a range of post-COVID-19 difficulties relating to the residents of Dhaka who received treatment at home and in the hospital. Performing physical activity and exposure to sunlight was positively associated with earlier recovery from covid-19 both in home and hospital care. People who initially received home-based treatment recovered earlier than those who were directly treated in hospitals. Age, level of education, physical exercise, practising preventive measures, and daily exposure to sunlight were significantly associated with place of treatment. Patients treated at home showed a reducing trend towards increased number of comorbidities, and home-to-hospital and directly hospital treated participants showed significant increasing trends with increased number of comorbidities. Post-COVID-19 complications like depression, chest pain, and digestive disorder were significantly associated with hospital-based treatment. While memory loss, loss of taste, cough, breathing difficulty, joint pain, fatigue, and muscle weakness were significantly associated with home-based treatment. The intake of carbohydrates, additional liquid, food supplements, and avoidance of junk foods were significantly associated with the place of treatment sought. The prescribed medicines used for participants were antipyretics, antihistamine, antiallergics, antibiotics, vitamins, and antivirals. Oxygen therapy was provided on requirement and some of the hospital-treated patients received ventilation and ICU support, and plasma therapy. The study also indicates that nursing at home in an early stage of COVID-19 infection is beneficial for patients and reduces the treatment costs. Further detailed studies are required to gauge the longevity of the complications and the impact on work-life balance as many of those affected were of working age. However, concerning the not well organised healthcare facilities in the country, this study aims to contribute to future research, in the field of healthcare delivery and capacity building based on the longer-term effects on morbidity due to Covid-19.

## Data Availability

The original contributions presented in the study are included in the article/supplementary materials, further inquiries can be directed to the corresponding author/s.
